# Holistic management of juvenile idiopathic arthritis across all ages: British Society for Rheumatology Guideline scope

**DOI:** 10.1093/rap/rkag052

**Published:** 2026-05-04

**Authors:** Sandrine Compeyrot-Lacassagne, Richard P Beesley, Lisa Bray, Angharad Bridges, Hema Chaplin, Coziana Ciurtin, Gavin Cleary, Samantha Cooray, Octavio Aragon Cuevas, Verna Cuthbert, Samundeeswari Deepak, Emily Earle, Andreia Ferreira, Lianne Kearsley-Fleet, Jo Gupta, Rebecca Houston, Ryan Malcolm Hum, Jenny H Humphreys, Hirushi S Jayasekera, Sarah Jones, Christine MacFadyen, Jennifer Nisbet, Visvalingam Arunath, Yeliz Prior, Valerie Rogers, Stephanie J W Shoop-Worrall, Ameenat Lola Solebo, Rosemary Waller, Luke Daniel Williamson, Debbie Wilson, Catherine Wright, Ethan S Sen, British Society for Rheumatology Guideline Steering Group, Edward Roddy, Devesh Mewar, Arvind Kaul, Coziana Ciurtin, Sandrine Compeyrot-Lacassagne, Anoop Kuttikat, Emma Williams, Abhishek Abhishek, Christopher Joyce, Karen Merrison, Emmandeep Dhillon, Claire Jones, Emily Rose-Parfitt, Hirushi Jayasekera, Pratyasha Saha

**Affiliations:** Department of Paediatric Rheumatology and NIHR Biomedical Research Centre, Great Ormond Street Hospital for Children NHS Foundation Trust, London, UK; Expert by Experience, Tonbridge, UK; Juvenile Arthritis Research, Tonbridge, UK; Department of Paediatrics, University Hospitals Sussex, St Richard’s Hospital, Chichester, UK; Expert by Experience, London, UK; Health Psychology Section, Institute of Psychiatry, Psychology and Neuroscience, King’s College London, London, UK; School of Health and Society, Centre for Applied Health Research, University of Salford, Salford, UK; Centre for Adolescent Rheumatology, Department of Ageing, Rheumatology and Regenerative Medicine, Division of Medicine, University College London, London, UK; Rheumatology Department, Alder Hey Children’s NHS Foundation Trust, Liverpool, UK; Department of Paediatric Rheumatology and NIHR Biomedical Research Centre, Great Ormond Street Hospital for Children NHS Foundation Trust, London, UK; Rheumatology Department, Alder Hey Children’s NHS Foundation Trust, Liverpool, UK; Pharmacy Department, Alder Hey Children’s NHS Foundation Trust, Liverpool, UK; Paediatric Rheumatology, Royal Manchester Children’s Hospital, Manchester, UK; Paediatric & Adolescent Rheumatology, Nottingham University Hospitals NHS Trust, Nottingham, UK; Expert by Experience, London, UK; CCAA—Kids With Arthritis, London, UK; Adult Rheumatology, University Hospitals Bristol and Weston NHS Foundation Trust, Bristol, UK; Children and Young Person’s Rheumatology Research Programme, Centre for Epidemiology Versus Arthritis, Centre for Musculoskeletal Research, University of Manchester, Manchester Academic Health Sciences Centre, Manchester, UK; Centre for Adolescent Rheumatology, Department of Ageing, Rheumatology and Regenerative Medicine, Division of Medicine, University College London, London, UK; Pharmacy Department, Robert Jones & Agnes Hunt NHSFT, Gobowen, UK; Rheumatology, Manchester University NHS Foundation Trust, Manchester, UK; Manchester Biomedical Research Centre, National Institute for Health and Care Research, Manchester, UK; Manchester Biomedical Research Centre, National Institute for Health and Care Research, Manchester, UK; Centre for Musculoskeletal Research, University of Manchester, Manchester, UK; Rheumatology Department, Shrewsbury and Telford Hospital NHS Trust, Shrewsbury, UK; General Internal Medicine, Shrewsbury and Telford Hospital NHS Trust, Shrewsbury, UK; Paediatric Rheumatology, Cardiff and Vale University Health Board, Cardiff, UK; Rheumatology Department, Great Western NHS Foundation Trust, Swindon, UK; Department of Rheumatology, Royal National Hospital for Rheumatic Diseases, Bath, UK; Paediatric Rheumatology, Royal Manchester Children’s Hospital, Manchester, UK; General Paediatrics, Manchester University NHS Foundation Trust, Manchester, UK; Postgraduate Institute of Medicine, University of Colombo, Colombo, Sri Lanka; Centre for Human Movement and Rehabilitation, School of Health and Society, University of Salford, Salford, UK; Paediatric Rheumatology, University Hospitals Bristol and Weston NHS Foundation Trust, Bristol, UK; Bath Centre for Pain Services, Royal United Hospitals, Bath, UK; Children and Young Person’s Rheumatology Research Programme, Centre for Epidemiology Versus Arthritis, Centre for Musculoskeletal Research, University of Manchester, Manchester Academic Health Sciences Centre, Manchester, UK; Department of Paediatric Rheumatology and NIHR Biomedical Research Centre, Great Ormond Street Hospital for Children NHS Foundation Trust, London, UK; Great Ormond Street Institute of Child Health, University College London, London, UK; Rheumatology Department, Great Western NHS Foundation Trust, Swindon, UK; Rheumatology Department, Barts Health NHS Trust, London, UK; Expert by Experience, Rickmansworth, UK; Inflammatory Arthritis UK, Rickmansworth, UK; Expert by Experience, Belfast, UK; Young People and Families Service, Arthritis UK, Belfast, UK; Department of Paediatric Rheumatology, Great North Children’s Hospital, Newcastle upon Tyne Hospitals NHS Foundation Trust, Newcastle upon Tyne, UK; Faculty of Medical Sciences, Newcastle University, Newcastle, UK

**Keywords:** Juvenile Idiopathic Arthritis, Holistic management, Life course, Guideline scope

## Abstract

What does this mean for patients?

Juvenile idiopathic arthritis (JIA) is the most common type of arthritis that starts before the age of 16 years. Many children and young people (CYP) continue to be affected by JIA and may need to be on treatment when they are adults. Most CYP with JIA have swollen, stiff and painful joints for at least 6 weeks. JIA can also affect other parts of the body. This includes the skin, the places where tendons join bones (called entheses) and sometimes internal organs. Some young people may also develop inflammation in their eyes, called uveitis. If this is not treated, it can affect eyesight and could lead to sight loss. People with JIA often need medicines to control inflammation. They are usually cared for by a team of healthcare professionals called a multidisciplinary team. This team may include doctors, nurses, pharmacists, physiotherapists, occupational therapists, podiatrists, psychologists and youth workers. They work together to help manage symptoms and prevent long-term damage. The British Society for Rheumatology (BSR) is the main organisation in the UK for healthcare professionals who care for people with JIA. BSR writes guidelines that help healthcare teams provide high-quality care. These guidelines are written with input from experts and people with lived experience. They help make sure that everyone receives good care wherever they live, including during the move from child to adult services. This article explains how BSR will develop a new guideline to diagnose, treat and monitor children, young people and adults with JIA. The guideline will follow the BSR protocol for creating clinical guidelines.

## Introduction

JIA is the most common childhood-onset inflammatory rheumatic disease, with an age-standardised prevalence of 43.5–70.2 per 100 000 [1]. Although the onset of disease is before the age of 16 years, JIA can affect people <1 year of age, with many having symptoms and requiring ongoing treatment throughout adulthood [2, 3]. JIA encompasses a heterogeneous group of related conditions that are often distinct from adult-onset arthritides. In a UK study, >60% of people diagnosed with JIA in childhood continued to receive specialist care (rheumatology or ophthalmology) beyond the age of 18 years [4]. In many cases, when young people with JIA transfer to adult care their diagnosis is inappropriately reclassified as an adult-onset disease such as RA [5]. This can lead to the use of disease activity measures that are not validated for JIA [6].

The current classification of JIA is based on the ILAR system, which groups people into seven categories: persistent or extended oligoarticular, RF-negative polyarticular, RF-positive polyarticular, psoriatic, enthesitis-related arthritis (ERA), systemic and undifferentiated JIA [7]. For the purpose of clinical trials and treatment pathways, the term polyarticular-course JIA is used to describe any ILAR category in which five or more joints are involved. Childhood arthritis can also occur in association with IBD, coeliac disease, IgA deficiency, type 1 diabetes [8], trisomy 21 (Down syndrome) [9] and cystic fibrosis, among others.

Systemic JIA is also referred to as Still’s disease and is considered equivalent to adult-onset Still’s disease [10]. Systemic JIA has the potential to affect multiple organ systems, particularly if complicated by macrophage activation syndrome (MAS), a type of secondary haemophagocytic lymphohistiocytosis. There are clinical and underlying pathogenesis similarities between RF-positive polyarticular JIA and RA in adults, between ERA and axial SpA, between IBD-related arthritis and enteropathic axial and peripheral arthritis, and between psoriatic JIA and PsA in adults [11]. There is also a group of people with ANA-positive persistent or extended oligoarticular and RF-negative polyarticular JIA and a high frequency of uveitis where there is no directly comparable adult disease [12].

The most common extra-articular manifestation of JIA is uveitis. It affects 20–25% of people with JIA [13]. Chronic anterior uveitis is usually asymptomatic and detected only by regular ocular examination as recommended by the 2006 guideline for screening for uveitis in JIA, produced jointly by the Royal College of Ophthalmologists and the British Society for Paediatric and Adolescent Rheumatology (BSPAR) [14]. Risk factors for development of uveitis are female gender, onset of arthritis at <7 years of age, oligoarticular pattern and ANA positivity. Uveitis is a serious complication of JIA that can be sight threatening. It is frequently bilateral and can remain active into adulthood. All categories of JIA may affect people’s joints and eyes, although uveitis is rarer in systemic and RF-positive polyarticular subtypes [4]. However, ERA-/SpA-associated uveitis typically presents as acute, symptomatic, unilateral anterior uveitis with recurrent flares, strongly associated with HLA-B27 and more common in older children and adolescents. It mirrors the phenotype seen in adult SpA and may persist into adulthood [15].

JIA and its treatment can impact physical function, social development, including participation in education and employment, and mental health [16, 17]. The approach to management of JIA should be holistic across the life course from infancy to adulthood and should involve the full multidisciplinary team (MDT).

## Why is the guideline needed?

This will be the first BSR guideline on the management of JIA and the first guideline internationally to take a whole-life-course approach and include recommendations regarding non-pharmacological management. A National Confidential Enquiry into Patient Outcome and Death (NCEPOD) report and the Getting It Right First Time (GIRFT) Paediatric Rheumatology report highlighted that there is no standardised, UK-wide guideline for the management of JIA [18, 19]. Development of the current guideline will address this gap. The report highlighted key recommendations, including access to intra-articular steroid injections, MDT care (including physiotherapy, occupational therapy, pain, podiatry and psychology services), patient and family education about JIA and its treatment and holistic and developmentally appropriate care across all ages.

The pharmacological management of JIA in England is currently based on the National Health Service (NHS) England clinical commissioning policy statement and flow chart published in 2015 that provides recommendations on treatment up to the age of 18 years [20]. There have been numerous clinical trials of biologic and targeted synthetic DMARDs (b/tsDMARDs) since 2015. An evidence-based update of this treatment pathway, which is applicable across all four nations of the UK, is needed. However, there have been only a few head-to-head comparison clinical trials in JIA to inform treatment sequencing. Where appropriate, we may draw on treatment pathways for the corresponding adult disease phenotypes to support the evaluation of evidence in JIA.

The screening process for JIA-associated uveitis is based on guidelines from 2006 [14]. There is a need to review more recent evidence to determine whether these guidelines remain valid. Only adalimumab is currently approved by NHS England for treatment of JIA-associated uveitis and a review of the evidence for other b/tsDMARDs is required to extend equitable access to people with uveitis that has not responded fully to adalimumab or for whom this drug is not suitable.

There is evidence available on developmentally appropriate care and a successful transition from paediatric into adult care [21–23]. This should lead to a proposed evidence-based transition pathway for people with JIA.

Quality improvement for JIA is embedded in the ethos of paediatric rheumatology teams across the UK. Implementing the recommendations from this guideline will require a collaborative approach, supported by improvement methodologies underpinning the BSR/GIRFT JIA Learn shared learning model [24] and recommendations arising from the GIRFT paediatric rheumatology report [19]. This approach aims to ensure equitable access to care and improved outcomes and experiences for people with JIA, regardless of their age, geographical location and socio-economic status.

The guideline will be in line with the three shifts described in the new NHS 10-year plan ‘Fit for the future’: hospital to community, analogue to digital and sickness to prevention [25]. This new strategic plan, launched in July 2025, will shape care delivery in the UK for the next 10 years. Following recent publications and the GIRFT paediatric rheumatology review, factors driving potential health inequalities should be considered in this guideline.

## Current practice

JIA is a chronic rheumatic condition with onset in childhood. The aim of treatment is to achieve complete remission of arthritis, uveitis and other extra-articular features (enthesitis, psoriasis, IBD), including systemic features [in systemic JIA (sJIA)] and complications (MAS); to avoid any joint, ocular or organ damage; to restore and maintain full physical activity with no functional impairment; to optimise quality of life and to avoid side effects of any treatments.

Current pharmacological treatment generally follows a stepwise pathway based on NHS England recommendations from 2015 [20]. In oligoarticular JIA, treatment usually starts with glucocorticoid intra-articular injections. In polyarticular or systemic JIA, induction treatment will often require systemic glucocorticoids. If a person has MAS associated with sJIA, multispecialty management and treatment will be required [26]. If methotrexate is not effective in controlling disease, or is not tolerated, the next step in treatment is a bDMARD. People with JIA who have axial or sacroiliac joint involvement or MAS unresponsive to steroids should be treated directly with a bDMARD without the need for prior methotrexate.

Treatment in England is governed by the NHS England pathway, while health services in Wales, Scotland and Northern Ireland have independent pathways.

Multiple b/tsDMARDs have undergone trials in children with JIA and are licenced, but not all are routinely funded in all nations of the UK. The treatments include bDMARDs directed against cytokines, cytokine receptors and cell surface proteins (TNF, IL-6 receptor, IL-1, cytotoxic T-lymphocyte associated protein 4, IL-17, IL-12/23, IL-23 and CD20) and tsDMARDs (Janus kinase inhibitors).

Access to bDMARD treatment specifically for JIA-associated uveitis is limited in England to the single anti-TNF adalimumab. The 2010 BSPAR/Arthritis and Musculoskeletal Alliance standards of care set out key recommendations for the management of JIA, including ophthalmology review within 6 weeks of diagnosis, education about JIA, training on treatment provided by appropriately qualified nurse specialists and access to the full MDT [27, 28].

## Who the guideline is for

This guideline is for all healthcare practitioners involved in the management of JIA. This includes, but is not restricted to, paediatricians with an interest in rheumatology, paediatric rheumatologists, adolescent rheumatologists, adult rheumatologists, clinical researchers, physiotherapists, occupational therapists, psychologists, podiatrists, specialist nurses and pharmacists involved in the care of people with JIA. The guideline is also for people with lived experience of JIA and their families, caregivers and support networks. It is also for NHS managers, healthcare commissioners and other stakeholders such as patient organisations.

## Equality considerations

Although the onset of JIA is in childhood, people of all ages can be affected by the condition. It occurs in all genders and ethnicities. Socio-economic, language, cultural and geographic barriers need to be considered and addressed to ensure equitable and optimal care of people with JIA. Access to drug treatments for JIA varies between the four nations of the UK. This guideline will make recommendations based on the scientific evidence, irrespective of whether funding is approved in one or more of the UK nations. Recent analysis has identified regional variations in access and funding, with inconsistency of NHS care affecting the most vulnerable [29, 30]. Recommendations will be made based on relevant published evidence to create a framework to tackle health inequalities in the management of JIA.

## Target clinical population

The guideline will cover people of all ages with JIA, including all ILAR categories and JIA-associated uveitis. It will also include IBD-associated arthritis and Down syndrome arthritis.

## Setting

Settings that will be covered include JIA in hospital-based settings, including secondary care rheumatology and tertiary hospital paediatric rheumatology, and in shared care with primary health services within the UK NHS.

## What will the guideline cover?

A survey was conducted to obtain consensus on the content of the guideline. The questions were based on the proposed scope and population, intervention, comparison and outcome (PICO) and agreed among the JIA guideline working group (GWG) members. One survey was directed to health professionals while a second one was developed by the experts by experience in the GWG and distributed to people with JIA and their families, caregivers and support networks. Responses were received from 127 health professionals and 37 people with JIA or their families. These responses were considered by the GWG in the process of developing the guideline scope.

The GWG will evaluate the most up-to-date evidence relating to the following aspects of JIA across all ages: screening and diagnosis of comorbidities associated with JIA, including uveitis; use of imaging for monitoring of disease activity and damage, and to aid treatment decisions; intra-articular steroid injections; non-pharmacological management of JIA, including physiotherapy, occupational therapy and psychology; pharmacological management of JIA and associated uveitis, including evidence for treat-to-target approaches and therapeutic drug monitoring; developmentally appropriate care and transition from paediatric to adult rheumatology services; and holistic care, including the interaction between JIA and education/employment, mental health, neurodiversity, growth and puberty

Further details relating to the scope of these topics are shown in Table 1.

**Table:** **Table 1** Key topics that will be included in the guideline for holistic management of JIA across all ages.

Screening of comorbidities in JIA	Screening of comorbidities (uveitis, IBD, psoriasis, coeliac disease, type 1 diabetes, IgA deficiency, growth impairment, puberty delay, bone density, hypercholesterolaemia, pain, mental health problems, psychosocial issues, neurodiversity, overweight/obesity and others) and monitoring during the disease
Role of imaging in JIA	Assessment of disease activity and damage using X-ray, ultrasound and MRI at diagnosis and during monitoring (including definition of remission) in CYP and adults diagnosed with JIA. This includes imaging of the spine, sacroiliac joints, temporomandibular joints and entheses
Intra-articular steroid injections in the management of JIA	How to use intra-articular steroid injections (timing, Entonox/general anaesthetic/distraction techniques, stand-alone treatment/adjuvant treatment) Choice of drug and efficacy (triamcinolone hexacetonide/acetonide), dosing and contraindications Role of imaging to guide injection Monitoring of side effects (local and systemic) Maximum number of joint injections (how many at one time, how frequently) individualised to the person based on previous experience in the management of CYP and adults with JIA
Non-pharmacological management of JIA	Evidence-based non-pharmacological MDT interventions examining impact on pain, fatigue, function, quality of life, engagement, concordance and overall outcome: Physiotherapy—exercise program and hydrotherapy Occupational therapy—splints, school adjustments Podiatry—foot health Education and training of people with JIA and their families/caregivers by clinical nurse specialists and -s with age-appropriate counselling, informed and shared decision making Psychology—mental health support, treatment concordance, pain/fatigue management, neurodiversity-informed approaches to communication and care Nutrition Peer support for people with JIA arranged by the MDT or third sector
Pharmacological management of JIA	Evidence-based pharmacological interventions, including NSAIDs, csDMARDs, bDMARDs and tsDMARDs with a timely escalation pathway and tapering strategies using outcome measures and based on classification criteria (in particular ILAR), medications in combination, use of biosimilars and use of bone marrow transplant Use of steroid, including doses, route of administration and side effects (and how to reduce these) Treat-to-target approaches and biomarker stratification strategies Therapeutic drug monitoring Definition of remission (including using biomarkers and imaging)
Quality improvement and clinical research in JIA	Learning network (with collection of minimal data set to assess outcomes allowing benchmarking across the four nations, creation of a formal network structure, experience sharing) Contribution to registries to continue learning from data, including linkage of paediatric and adult datasets through national trusted research environments to explore long-term outcomes of JIA into adulthood Contribution to clinical research Cost-effectiveness of intervention and commissioning underpinning access to medications across the four nations Evidence-based suggestions to improve data capture and reporting regarding disease activity and medication prescribing Digital health strategy to improve care through the National Centre for Child Health Technology Evidence-based care pathways within a regional network describing the role of stakeholders to support community-based care, build effective collaboration, reduce inequalities and improve care
Developmentally appropriate care in JIA	Developmentally appropriate care for CYP and adults with JIA, including Planning of transition from paediatric to adult rheumatology Presence of a chaperone Guideline for parents’/guardians’ attendance Timing of appointments Lifestyle counselling Role of youth workers Empowerment of people with JIA Developmentally appropriate education with injection training Mental health evaluation Monitoring of growth, including weight This guideline will summarise current and relevant evidence in developmentally appropriate care, including systematic reviews and national recommendations
Healthcare inequality in JIA across the UK	Raise awareness of JIA to improve early recognition Language and neurodiversity barriers Sociodemographic circumstances Health professionals’ resources in primary and secondary care to provide patient-centred local care Cultural access support Inequality of medication access in CYP and adults with JIA, including the time between the decision to start a new treatment and the first dose being given This guideline will summarise current and relevant evidence in healthcare inequality, including systematic reviews and national recommendations

## Areas that will not be covered

This guideline will not cover forms of arthritis not diagnosed as JIA, such as septic arthritis or arthritis as part of other autoimmune/inflammatory diseases, including SLE and JDM (although IBD-associated arthritis and Down syndrome arthritis will be included) ; uveitis associated with conditions other than JIA (such as sarcoidosis, tubulointerstitial nephritis and uveitis, infection-associated uveitis) and isolated idiopathic uveitis; referral pathways for JIA (which will be covered in the ‘Hot, swollen or stiff joints in children and young people’ guideline) [31]; pretreatment screening, monitoring and safety of conventional synthetic DMARDs (csDMARDs) (which is covered in another BSR guideline) [32]; pre-treatment screening, monitoring and safety of bDMARDs and tsDMARDs (which is covered in another BSR guideline) [33, 34]; safety of DMARDs in pregnancy (which is covered in another BSR guideline) [35]; pain management in people with JIA (which is covered in another BSR guideline) [36] and joint repair/replacement and other surgical management of joint disease in JIA.

## Related guidelines and recommendations

Related guidelines and recommendations include the following:

- 2006 Guidelines for Screening for Uveitis in Juvenile Idiopathic Arthritis, produced jointly by the BSPAR and the Royal College of Ophthalmology [14]
- 2010 BSPAR Standards of Care for children and young people with juvenile idiopathic arthritis [27]
- 2015 National Institute for Health and Care Excellence evidence-based recommendations on abatacept, adalimumab, etanercept and tocilizumab for treating juvenile idiopathic arthritis in children, young people and adults [37]
- 2019 American College of Rheumatology/Arthritis Foundation Guideline for the Treatment of Juvenile Idiopathic Arthritis: Therapeutic Approaches for Non-Systemic Polyarthritis, Sacroiliitis, and Enthesitis [38]
- 2019 American College of Rheumatology/Arthritis Foundation Guideline for the Screening, Monitoring, and Treatment of Juvenile Idiopathic Arthritis-Associated Uveitis [39]
- 2021 American College of Rheumatology Guideline for the Treatment of Juvenile Idiopathic Arthritis: Therapeutic Approaches for Oligoarthritis, Temporomandibular Joint Arthritis, and Systemic Juvenile Idiopathic Arthritis [40]
- 2021 American College of Rheumatology Guideline for the Treatment of Juvenile Idiopathic Arthritis: Recommendations for Nonpharmacologic Therapies, Medication Monitoring, Immunizations, and Imaging [41]
- 2024 EULAR/Paediatric Rheumatology European Society (PReS) recommendations for the diagnosis and management of Still’s disease, comprising systemic juvenile idiopathic arthritis and adult-onset Still’s disease [42]
- 2025 French protocol for the diagnosis and management of juvenile idiopathic arthritis including paediatric-onset Still’s disease [43]
- 2025 treatment of polyarticular juvenile idiopathic arthritis in Latin America: recommendations from the Pan-American League of Associations for Rheumatology [44]
- 2025 recommendations for the treatment of juvenile idiopathic arthritis with oligoarthritis or polyarthritis from the 2024 update of the Japan College of Rheumatology Clinical Practice Guidelines for the management of rheumatoid arthritis including juvenile idiopathic arthritis with oligoarthritis or polyarthritis—secondary publication [45]
- 2025 Taiwan guideline for the diagnosis and management of juvenile idiopathic arthritis: consensus statement of the Taiwan Academy of Pediatric Allergy, Asthma and Immunology [46]
- 2025 African guidelines for diagnosis and management of polyarticular juvenile idiopathic arthritis: PAFLAR initiative [47]
- 2025 Pediatric Society of the African League Against Rheumatism juvenile idiopathic arthritis recommendations for enthesitis-related arthritis and juvenile psoriatic arthritis [48]
- 2025 National Confidential Enquiry into Patient Outcome and Death: a review of the quality of care provided to children and young adults with juvenile idiopathic arthritis [18]
- 2025 paediatric rheumatology national GIRFT report [19]
- British Society for Rheumatology guideline: Hot, swollen or stiff joints in children and young people: British Society for Rheumatology guideline scope [31]
- 2025 British Society for Rheumatology guideline for the prescription and monitoring of conventional synthetic disease-modifying anti-rheumatic drugs [32]
- British Society for Rheumatology biologic DMARD safety guidelines in inflammatory arthritis [33, 34]
- Pain management in people with inflammatory arthritis: British Society for Rheumatology guideline scope [36].

## Methods

The guideline development will follow the BSR ‘Creating Clinical Guidelines’ protocol version 6.0 (October 2025) [49]. The methodology will include

- Systematic evidence searches and, where relevant and feasible, evidence synthesis by an independent evidence synthesis team.
- Expert committee review: A panel of clinical experts, academics and experts by experience (people living with JIA and family representatives) will review the gathered evidence.
- Public and professional consultation: A draft will be available for consultation among health professionals and patient/family groups to ensure it meets clinical needs.
- Final revisions and publishing: Incorporate feedback and finalise the guideline.

This guideline will be created by a working group consisting of healthcare professionals and experts by experience: young people and their parents/caregivers who have experience living with JIA. The healthcare professionals include paediatric rheumatologists, paediatricians with an interest in rheumatology, adolescent and adult rheumatologists, a paediatric ophthalmologist, specialist nurses, physiotherapists, occupational therapists, pharmacists, psychologists and epidemiologists.

Evidence reviews will be conducted by an independent evidence synthesis team to inform guideline recommendations. Each review will address one or more key topics (Table 1) and associated questions and involve systematic searches of relevant sources, e.g. bibliographic databases or grey literature, and screening of results with reference to predefined selection criteria. Where appropriate, full systematic reviews will be undertaken, e.g. for pharmacological and non-pharmacological treatments. This will additionally include data extraction using a pretested form and critical appraisal of each study to assess study limitations and risk of bias. This evidence will then be synthesised narratively with the quality of evidence for prioritised outcomes assessed using a Grading of Recommendations Assessment, Development, and Evaluation–informed approach [50].

## Structured questions using PICO or SPIDER (sample size, phenomenon of interest, study design, evaluation and research type) frameworks for evidence reviews

The PICO and SPIDER frameworks are the most appropriate frameworks to assist in structuring research questions in the JIA guideline [51]. The PICO framework (or adapted PICO framework such as PI-O) has been used for evidence-based clinical practice (Table 2) and the SPIDER framework for qualitative research and mixed-methods research (Table 3).

**Table:** **Table 2** PICO framework applied to some JIA guideline topics.

PICO topics	Population	Intervention	Comparison	Outcome^a^
Imaging	JIA	Diagnostic investigations: X-ray, ultrasound or MRI (with or without contrast)	Clinical assessment only	Disease activity Damage Comorbidities Treatment decision-making escalation and weaning
Uveitis screening (length and frequency)	JIA	Screening based on ILAR criteria/age	Absence of screening or different screening protocols	Early diagnosis (detection rate or time to diagnosis) Damage-free uveitis
Joint injection	JIA	Corticosteroid injection, as listed in Table 1	No additional corticosteroid injection Ultrasound-guided *vs* unguided Triamcinolone *vs* hexacetonide	Complications and side effects Role in the treatment pathway
Non-pharmacological management	JIA	Non-pharmacological MDT interventions, as listed in Table 1	Only pharmacological intervention Comparison between different non-pharmacological interventions Waiting list or sham/attention control	Outcome improvement: pain, function, quality of life education, engagement adherence
Pharmacological management	JIA	NSAIDs or DMARDs, as listed in Table 1	Placebo, no additional drug or comparison between different types of drugs. Evidence-based escalation pathway based on ILAR classification and presence of uveitis	Early damage-free remission Quality of life Cost-effectiveness and side effects of corticosteroids Adherence
Non-pharmacological monitoring	JIA	Monitoring using digital health, biomarkers, treat-to-target approaches, therapeutic drug monitoring, patient-reported outcome measures	Clinical and radiological monitoring only	Optimisation of care pathway Cost-effectiveness Quality of life

a Design: randomized controlled trials, non-randomised controlled trials, other comparative observational designs.

**Table:** **Table 3** SPIDER framework applied to three JIA guideline topics.

SPIDER topics	Sample	Phenomenon of interest	Design	Evaluation	Research
Screening for comorbidities	JIA	IBD, chronic non-bacterial osteomyelitis, psoriasis, type 1 or 2 diabetes, IgA deficiency, growth, bone health, hypercholesterolaemia, pain, psychosocial issues, obesity	Observational designs: cross-sectional studies, longitudinal cohorts	Disease outcome Additional needs Experience	Quantitative studies
Developmentally appropriate care	JIA all age	Parent/guardian attendance Lifestyle counselling Empowerment Readiness evaluation Education with injection training Mental health evaluation	Qualitative designs and observational studies (e.g. surveys)	Disease outcome Experience of care Attendance Concordance	Mixed methods
Health inequalities	JIA	Awareness of JIA language barriers Support based on sociodemographic circumstances Patient-centred local care Inequality of medication access	Qualitative designs and observational studies (e.g. audits, surveys)	Variability in access, uptake or delivery of care across population groups (ethnicity, geography, deprivation) Disease outcome	Mixed methods

We are developing this guideline to ensure that people with JIA will receive updated holistic MDT care with a treatment pathway updated with the latest evidence. This guideline will follow the recommendations of the NCEPOD JIA national audit and the GIRFT paediatric rheumatology national review. We plan to issue recommendations that may contribute to future audits, benchmarking, quality improvement and research. We anticipate that the guideline will be in a format that facilitates its use in clinical practice, with a focus on graphical abstracts and a breakdown of information based on topics and on health professionals’ use in routine care.

## Timeline for publication of guideline

The guideline is expected to be published in 2027.

## GWG

The multidisciplinary GWG comprises the following members from across the UK: Sandrine Compeyrot-Lacassagne (co-chair, consultant paediatric rheumatologist), Ethan S. Sen (co-chair, consultant paediatric rheumatologist), Richard P. Beesley (expert by experience), Lisa Bray (consultant in paediatrics and paediatric rheumatology), Angharad Bridges (expert by experience), Hema Chaplin [chartered psychologist (researcher)], Coziana Ciurtin (consultant in adolescent/young adult and adult rheumatology), Gavin Cleary (consultant paediatric rheumatologist), Samantha Cooray (specialist registrar in paediatric rheumatology), Octavio Aragon Cuevas (lead paediatric rheumatology pharmacist), Verna Cuthbert (advanced physiotherapist paediatric rheumatology), Samundeeswari Deepak (consultant paediatric rheumatologist), Emily Earle (expert by experience), Andreia Ferreira (lead clinical nurse specialist), Lianne Kearsley-Fleet (research fellow epidemiology), Jo Gupta (clinical nurse specialist), Rebecca Houston (lead pharmacist—rheumatology, bone health and clinical trials), Ryan Malcolm Hum (clinical PhD fellow and speciality registrar in rheumatology), Jenny H. Humphreys (senior clinical lecturer and honorary consultant rheumatologist), Hirushi S. Jayasekera (rheumatology and general internal medical registrar), Sarah Jones (clinical specialist physiotherapist in paediatric rheumatology), Christine MacFadyen (rheumatology registrar), Jennifer Nisbet (highly specialised paediatric occupational therapist), Visvalingam Arunath (senior clinical fellow), Yeliz Prior (occupational therapist), Valerie Rogers (consultant in paediatric rheumatology and chronic pain), Stephanie J. W. Shoop-Worrall (research fellow epidemiology), Ameenat Lola Solebo (consultant paediatric ophthalmologist), Rosemary Waller (consultant rheumatologist), Luke Daniel Williamson (consultant rheumatologist), Debbie Wilson (expert by experience), Catherine Wright (expert by experience).

## Supplementary Material

rkag052_Supplementary_Data

## Data Availability

No new data were generated or analysed in support of this article.
