# Supplementary material for: Holistic management of juvenile idiopathic arthritis across all ages: British Society for Rheumatology Guideline scope
Source: Rheumatol Adv Pract. 2026 May 4;10(2):rkag052. doi: 10.1093/rap/rkag052 (PMC13235788; doi:10.1093/rap/rkag052)
Supplement: rkag052_Supplementary_Data [file rkag052_supplementary_data.docx]

**Supplementary Data S1. British Society for Rheumatology Guideline Steering Group members**

Edward Roddy, Devesh Mewar, Arvind Kaul, Coziana Ciurtin, Sandrine Compeyrot-Lacassagne, Anoop Kuttikat, Emma Williams, Abhishek Abhishek, Christopher Joyce, Karen Merrison, Emmandeep Dhillon, Claire Jones, Emily Rose-Parfitt, Hirushi Jayasekera, Pratyasha Saha
